# IFN-γ stimulated dental follicle mesenchymal stem cells regulate activated lymphocyte response in rheumatoid arthritis patients in vitro

**DOI:** 10.3906/sag-1812-152

**Published:** 2019-12-16

**Authors:** Noushin ZIBANDEH, Deniz GENÇ, Nevsun İNANÇ, Haner DİRESKENELİ, Tunç AKKOÇ

**Affiliations:** 1 Department of Pediatric Allergy and Immunology, Marmara University, School of Medicine, Istanbul Turkey; 2 Division of Rheumatology, Marmara University, School of Medicine, Istanbul Turkey

**Keywords:** Inflammation, mesenchymal stem cells, rheumatoid arthritis

## Abstract

**Background/aim:**

Multipotent mesenchymal stem cells (MSCs) have been investigated in autoimmune diseases such as rheumatoid arthritis (RA) due to their immunomodulatory and regenerative properties. In this study, their immunosuppressive effects on peripheral blood mononuclear cells (PBMC) of RA patients were studied.

**Materials and methods:**

Dental follicle stem cells (DFSCs) were isolated from follicle tissue in the orofacial region. Characterization and multipotency analyses were performed. Lymphocytes were isolated from peripheral venous blood of RA patients (n = 5) and healthy individuals (n = 5). DFSCs were preincubated with IFN-γ for 48 h. PBMCs of RA patients and healthy individuals were separately cultured with or without DFSCs for 72 h. After culture period, lymphocyte proliferation and viability, the frequency of CD4+ CD25+FoxP3+ T regulatory cells, IL-10 and TNF-α levels in the culture supernatants were measured via flow cytometry.

**Results:**

Our results demonstrated that DFSCs suppressed proliferation of T lymphocytes by increasing the number of FoxP3 expressing CD4+CD25+ T regulatory cells and suppressed lymphocyte apoptosis in RA patients. Also, DFSCs reduced TNF-α cytokine secretion and upregulated IL-10 secreting cells.

**Conclusions:**

Such cells could potentially be a source for future immunomodulatory treatments of RA patients.

## 1. Introduction

Rheumatoid arthritis (RA) is a systemic autoimmune disease characterized by pain and stiffness of the joints and inflammation of the synovial membrane that may cause severe morbidity and mortality. Although the precise etiology of RA remains unclear, autoreactive T cells and inflammatory cytokines such as TNF-α, IL-1β, and IL-6 have a significant role in the pathogenesis of RA. Synovial inflammation and joint destruction are induced by the production of matrix metalloproteinase as well as the maturation and activation of osteoclasts (1–3). Mechanisms of joint destruction appear to be, at least in part, uncoupled from inflammation (4). Therefore, suppression of inflammation may not be sufficient to stop RA disease progression.

Biologic therapies inhibiting inflammation reduce the clinical symptoms and increase complete remission. Currently available biological agents for the management of RA are anticytokines (anti-TNFα, anti-IL-1β, or anti-IL-6), anticell therapies (anti-B cell, CTLA-4Ig), and signal inhibitors (JAK inhibitors). Although biologics are mostly effective, there is still an unmet clinical need for new approaches as patients’ refractory to current biologics are at risk of further cartilage and joint damage. Therefore, novel treatment strategies for both antiinflammation and bone regeneration are required (5–7).

Mesenchymal stem cells (MSCs) are multipotent cells that can be achieved from many tissues like bone marrow, adipose and dental tissues, and are able to differentiate into various lineages of cell types (8). MSCs originating from the orofacial region are easy to access and isolate (9). In addition to their differentiation potential and tissue regeneration, dental MSCs have been reported to regulate immune responses in autoimmune diseases. MSCs have potent immunosuppressive and antiinflammatory effects through direct cell-cell contact or by secreting soluble factors such as indoleamine 2,3-dioxygenase, TGF-β, prostaglandin E2 (PGE2), and nitric oxide synthase, and inhibit the activation, proliferation, and function of a variety of immune cells including T cells, B cells, natural killer (NK) cells, and antigen-presenting cells. Besides, they have the potential for clinical application in the repair of damaged tissue. FoxP3 expressing Treg cells play a significant role in controlling autoimmune responses and maintaining peripheral tolerance (10–11). Deficiencies in Treg cell function have been identified in autoimmune diseases including RA (12), psoriasis (13), and myasthenia gravis (14). Interferon-gamma (IFN-γ) is known to enhance the immunosuppressive properties of MSC. IFN-γ preactivated MSCs directly or indirectly regulate T cell responses by increasing or inducing MSC inhibitory factors (15).

In some studies of RA, patients data suggested that bone marrow or adipose-derived MSCs have the ability to regulate the immune functions by reducing the inflammatory Th1/Th17 responses and enhancing the protective regulatory T cell response (16,17). The recent evidence that MSCs with the capacity to differentiate into cartilage are available in joint tissues gives an opportunity for the therapeutic use of these cells (18). However, it is not exactly known whether MSCs have inflammatory or antiinflammatory effect in RA.

In the present study, we investigated the immunomodulatory effects of DFSCs on T cells in RA patients in vitro. Cytokine levels were also investigated to explain the regulatory effects of DFSCs on mononuclear cells in RA. This study provides basic data for further detailed investigation of DFSCs as a new and effective source for cell-based therapies in inflammatory disease.

## 2. Materials and methods

### 2.1. Ethics

This study was approved by the Ethics Committee of the Marmara University School of Medicine, Istanbul, Turkey (Protocol No: 09.2015.196/70737436–050.06.04). Written informed consent was obtained from all patients.

### 2.2. Isolation, characterization, and differentiation potential of DFSCs

Isolation of mesenchymal stem cells was performed as previously described (16). Briefly, dental follicle tissues were cut into 0.5 mm pieces and enzymatically digested with 3 mg/mL collagenase type I (Gibco, USA) for 45 min at 37 °C. Inactivation of the enzyme cell pellets was transferred to T-25 flasks containing 5 × 10^3^ cells/ cm^2^ and incubated at 5% CO_2_ atmosphere at 37 °C in culture medium composed of DMEM, 10% FBS, and 1% penicillin/streptomycin. Stem cells were detached with 0.25% trypsin-EDTA (Gibco, USA) and passaged until the third passage. Third passage cells were analyzed for surface antigen expressions. Cells were incubated with antibodies of positive markers for mesenchymal stem cells of human CD29, CD73, CD105, CD146, and negative markers CD14, CD34, CD20, and HLA-DR (BD Biosciences, USA) for 15 min at room temperature in the dark. Flow cytometry results were analyzed using BD FACS Calibur.

Osteogenic, adipogenic, and chondrogenic stimulants (StemPro) were added separately into seeded cell cultures to induce the differentiation process. The differentiation assay was performed in 6-well plates (5 × 10^4^ cell/well); differentiation media were prepared according to the manufacturer’s instructions and changed 3 times per week. After 14 days, the osteocytes, adipocytes, and chondrocytes were stained with alizarin red, oil red O, and alcian blue, respectively, and after 21 days the cell types were evaluated under a microscope.

### 2.3. Isolation of peripheral blood mononuclear cells (PBMC)

Peripheral blood was collected from 6 RA patients (2 men and 4 women). All patients met the American College of Rheumatology 1987 revised classification criteria for RA, and disease duration of patients was a minimum of 24 months. They had active disease and were not receiving corticosteroids and disease modifying antirheumatic drugs at the time of sample collection. In addition, peripheral blood was collected from 6 healthy individuals (3 men and 3 women) without an autoimmune or inflammatory disease as control group.

PBMC were isolated from heparinized peripheral blood samples by using Ficoll-Paque (GE Healthcare Bio-Sciences) density gradient solution, as previously described (14). The cells were cultured in RPMI (Gibco, USA) supplemented with 10% FBS and 1% penicillin/streptomycin with the addition of stimulatory agents.

### 2.4. Coculture of PBMC with DFSCs

DFSCs (5 × 10^4^/well in a 48-well plate) were plated 48 h prior to the addition of tenfold of lymphocytes in the culture medium. DFSCs and PBMCs (1:10) were cocultured for 72 h. The cultures were stimulated by using 0.5 μg/mL of CDmix (anti-CD3 and anti-CD28), IFN-𝛾(0.5 μg/mL, Millipore, CA, USA), Derp 1 (0.5 μg/mL), or Dexamethasone (10^–4^ M). Then, lymphocyte proliferation (carboxyfluorescein succinimidyl ester, CFSE), apoptosis (Annexin V/PI), CD4+CD25+FoxP3+ Treg ratios from PBMC, and cytokine levels from culture supernatants were analyzed via flow cytometry.

### 2.5. CFSE assay and evaluation of lymphocyte proliferation

The proliferation of lymphocytes was evaluated by labeling PBMC with 10 μM carboxyfluorescein succinimidyl ester (CFSE) (Invitrogen, USA). The lymphocytes were cultured unstimulated or stimulated with anti-CD3/anti-CD28 (CDmix) in the presence and absence of DFSCs and analyzed for CFSE signaling via flow cytometry (FACS Calibur).

### 2.6. Detection of apoptosis of the lymphocytes by Annexin V/PI

After 72 h of incubation period, the apoptosis ratio of lymphocytes was quantified by using the Annexin V/PI kit (eBiosciences, USA) according to the manufacturer’s instructions. Flow cytometric analysis was performed by staining PBMC with anti-CD3 and anti-CD4 antibodies for Annexin V/PI detection.

### 2.7. CD4^+^CD25^+^FoxP3^+^ Tregulatory cell frequency

After 72 h of coculture, the CD4^+^CD25^+^FoxP3^+^ Treg lymphocytes were quantified using Human FoxP3 Buffer Kit (eBioscience, USA). The frequency of FoxP3 expressing regulatory T cells (CD4^+^CD25^+^FoxP3^+^) was analyzed in the cultured lymphocytes via flow cytometry. The kit included antihuman CD4 (FITC), antihuman CD25 (APC), and antihuman FoxP3 (PE) (eBioscience, USA).

### 2.8. Analysis of cytokine expression profiles

Supernatant from cultures was collected and stored at –80 °C until assayed. Samples were measured and analyzed for TNF-α and IL-10 cytokine levels by Cytokine Bead Array (CBA) kit (BD Biosciences, USA) according to the manufacturer’s protocol. Briefly, all the CBA kit contents and samples should be at room temperature for at least 15 min. Fifty μL of culture supernatants, 50 μL of capture beads, and 50 μL of detection reagent were added and incubated for 3 h. After incubation, samples were washed twice with cold PBS. Samples were acquired in a FACS Calibur flow cytometer (BD Biosciences) and analyzed using the FCAP Array v1.0.1 software (Soft Flow Inc.). Results were expressed as picograms per milliliter.

### 2.9. Statistical analysis

The statistical analysis was achieved by using GraphPad Prism 5 (GraphPad Software, La Jolla, CA, USA). Oneway analysis of variance (ANOVA) with Tukey’s multiple comparisons was used for multigroup comparisons, and a two-tailed unpaired Student’s t-test was used for comparisons between two groups; a P value of < 0.05 was considered statistically significant.

## 3. Results

### 3.1. Isolation, characterization, and differentiation of DFSCs

**Figure 1:**
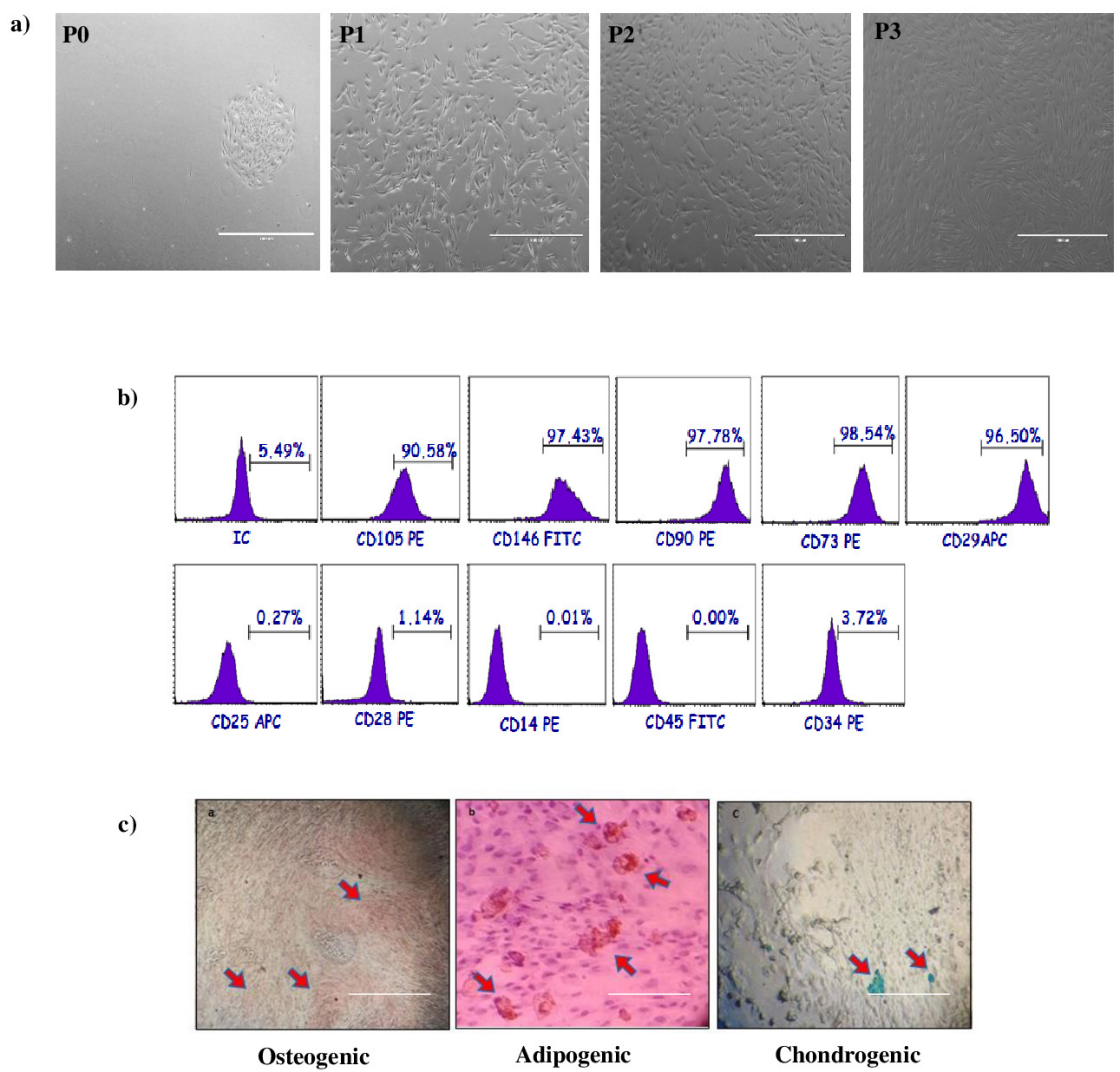
Morphological appearance, characterization, and multipotency of DFSCs. A) Morphology of DFSCs in P0, P1, P2, and P3 (magnification = 10×). B) Representative flow cytometry analysis of positive surface markers CD105, CD146, CD90, CD73, CD29, and negative surface markers CD25, CD28, CD14, CD45, CD34 for DFSCs at third passages. C) alizarin red staining of osteogenic induced DFSCs, oil red staining of adipogenic induced DFSCs, Alcian blue staining of chondrogenic induced DFSCs; scale bar = 1000 μm.

The MSCs were isolated from dental follicle tissues. Their proliferation gradually formed small colonies in 3 days. The MSCs reached 70% confluency in the primary culture 7 days after plating for the first passage. Most of the MSCs exhibited fibroblast-like morphology at the P3 passage (Figure 1a).

The MSCs were analyzed via flow cytometry. These cells exhibited positive staining for CD29, CD90, CD 146, CD73, and CD106, but were negative for CD34, CD45, CD14, CD28, and CD25 (Figure 1b). 

The MSCs differentiated into osteocytes, adipocytes, and chondrocytes. First, the osteogenic differentiation capability was investigated in vitro during a 28-day culture period in osteogenic induction medium. The MSCs were stained with alizarin red and the cells formed calcified bone nodule structures. Next, in vitro adipogenic differentiation capability was assessed by culturing the cells in adipogenic induction medium and staining with oil red O. Intracellular lipid droplets were observed in these cells. Chondrogenic differentiation capability was investigated in vitro following 14-day culture period in chondrogenic induction medium and cell differentiation into chondrocytes was confirmed with alcian blue staining that exhibited intracellular proteoglycans in those cells (Figure 1c).

### 3.2. DFSCs decreased proliferative response of lymphocytes in RA patients

**Figure 2:**
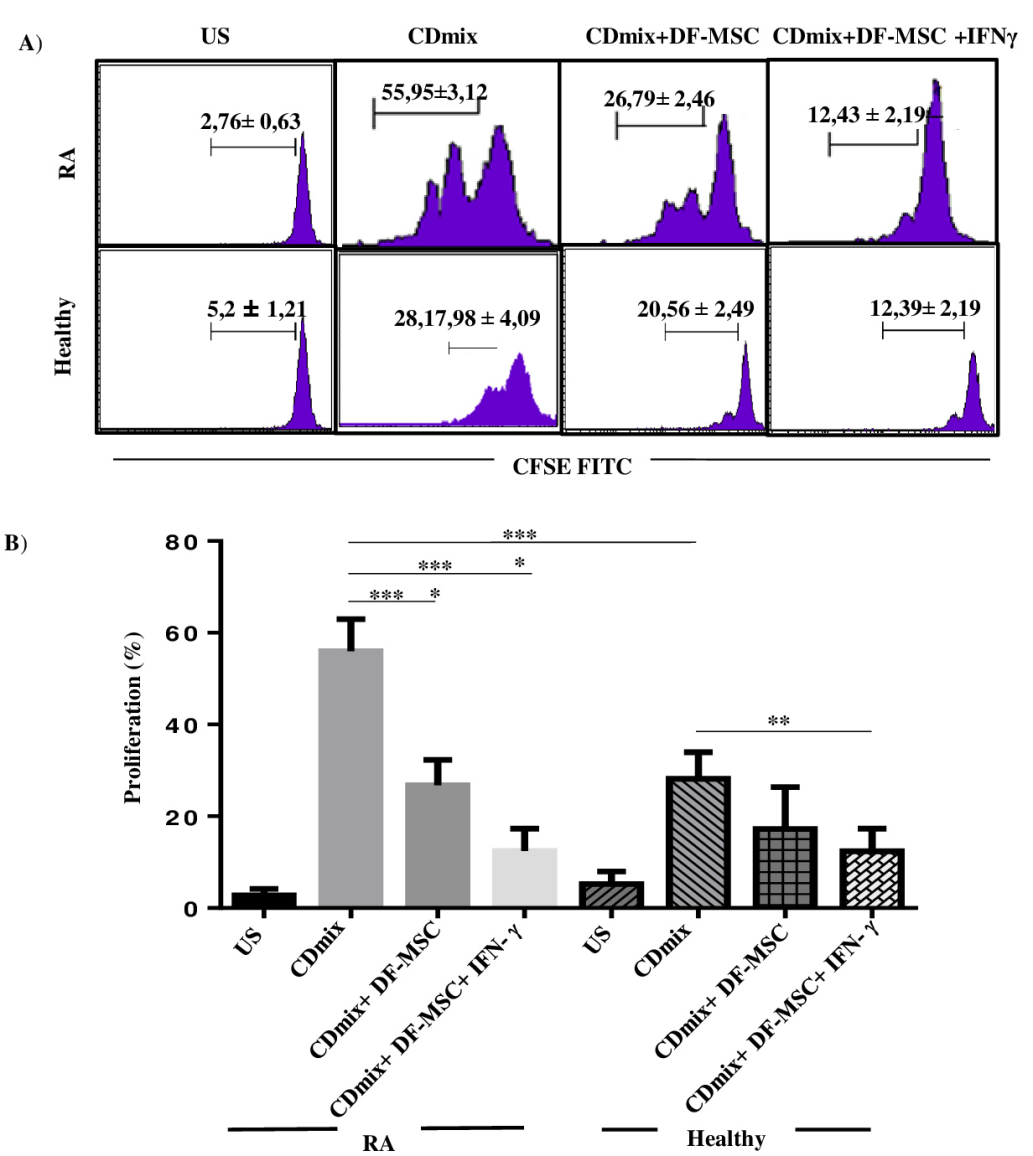
Inhibitory effect of DFSCs on the proliferation of lymphocytes as detected by CFSE. A) After 72 h of coculture, effect of DFSCs on the proliferation of lymphocytes displayed by flow cytometry. IFN-g stimulated DFSCs suppressed lymphocyte proliferation. B) Inhibitory effect of DFSCs on the proliferation of lymphocytes displayed statistically. *P < 0.05, **P < 0.01, ****P < 0.0001. Results are shown as mean ± SD.

We investigated the immunomodulatory effect of T lymphocytes in RA and healthy controls by CFSE cell labeling. Lymphocyte proliferation capacity was significantly higher in RA patients (55.95 ± 3.12) compared to healthy controls (30.98 ± 1.72; P < 0.001). DFSCs significantly suppressed the proliferation of lymphocytes in the cocultures of CDmix stimulated PBMCs in RA patients when compared with PBMC cultures alone (P < 0.001). IFN-γ stimulation of DFSCs further suppressed the lymphocyte proliferation in RA patients (12.43 ± 2.19) when compared with PBMC cultures alone (55.95 ± 3.12; P < 0.0001). In the presence of DFSCs, lymphocyte proliferation of healthy controls tended to decrease, but the difference was not significant (P > 0.05). IFN-γ stimulated DFSCs significantly suppressed lymphocyte proliferation capacity in healthy controls (12.39 ± 2.19) compared to PBMC cultures alone (28.17 ± 2.60; P < 0.01) (Figure 2).

### 3.3. DFSCs enhance the ratio of viable lymphocytes in RA patients

**Figure 3:**
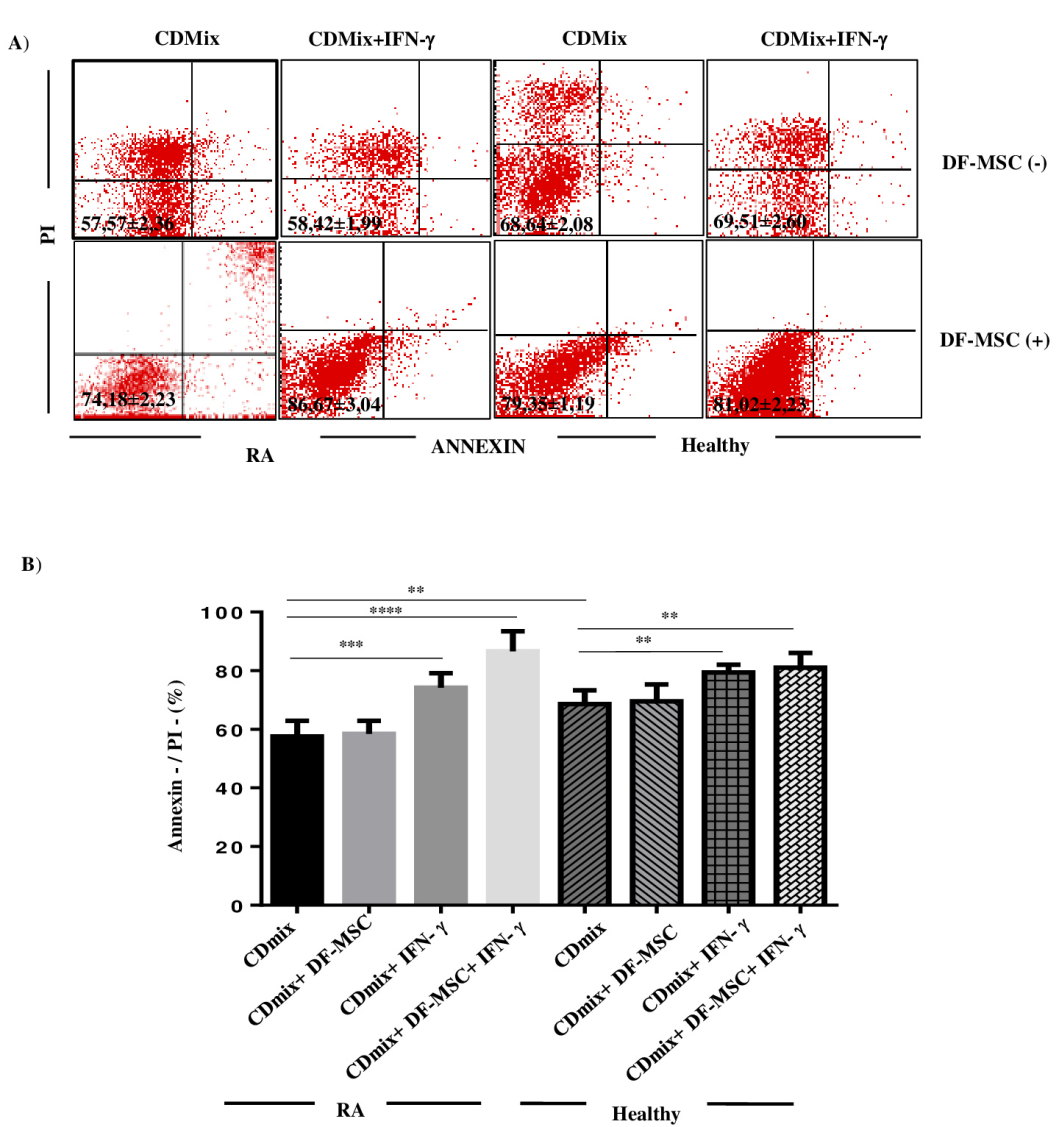
Cell survival analysis of T lymphocytes. A) Lymphocytes were stained with Annexin V (PE) and PI in order to analyze cell survival and apoptotic ratio of lymphocytes. Lower left quadrants (Annexin-/PI-) were evaluated for cell survival ratio. B) In statistical analysis, cell survival ratio of PBMC increased with DFSCs in RA and healthy control groups, and it is statistically significant (****P < 0.0001, P < 0.01, respectively). IFN-g enhanced antiapoptotic characteristic of DFSCs, especially in RA patients. *P < 0.05, **P < 0.01, ****P < 0.0001. Results are shown as mean ± SD.

To investigate the effect of DFSCs on cell viability of lymphocytes, Annexin V-/PI- ratio is determined on the surface of lymphocytes. The viable lymphocyte ratio stimulated with CDmix was lower in RA patients (57.57 ± 2.36) compared to healthy controls (68.64 ± 2.08, P < 0.01). DFSCs increased the cell viability of T lymphocytes in RA (74.18 ± 2.23) and healthy groups (79.35 ± 1.19), which was more prominent in RA patients compared to healthy controls (P < 0.001 and P < 0.01, respectively). IFN-γ stimulated DFSCs also increased antiapoptotic effect of DFSCs in RA patients (P < 0.0001) (Figure 3).

### 3.4. DFSCs increased CD4+CD25+FoxP3+ Treg cell ratio in RA patients

**Figure 4:**
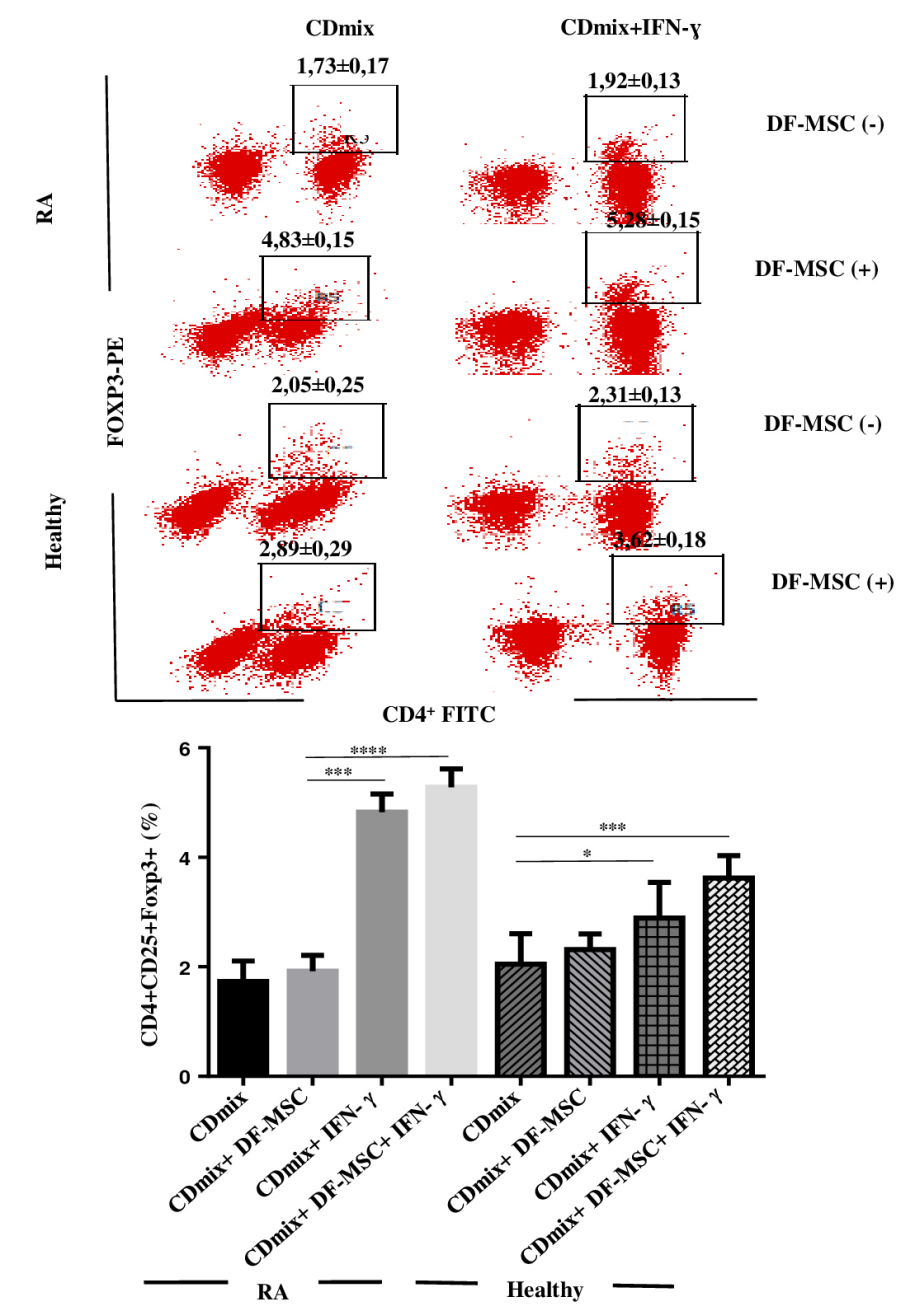
CD4+CD25+Foxp3+ analysis of T lymphocytes. A) Lymphocytes were stained with CD4+ (FITC), CD25+ (APC), and Foxp3+ (PE) in order to analyze the frequency of Treg cells. CD4+Foxp3+ cells were analyzed from CD25+ cells. B) In statistical analysis, CD4+ CD25+Foxp3+ ratio of PBMC increased with DFSCs in RA and healthy control groups, and it is statistically significant (***P < 0.001, P < 0.05, respectively). IFN-g stimulated DFSCs enhanced frequency of CD4+CD25+Foxp3+ cell ratio, especially in RA patients, compared to unstimulated DFSCs. *P < 0.05, ***P < 0.001, ****P < 0.0001. Results are shown as mean ± SD.

We studied the effects of DFSCs on the Treg cell ratio in RA patients’ PBMCs. CD4+CD25+FoxP3+ Treg cell ratio was significantly lower in unstimulated, CDmix stimulated PBMC cultures compared to healthy individuals (P < 0.05). DFSCs increased the CD4+CD25+FoxP3+ Treg cell ratio significantly in unstimulated, CDmix cocultures compared to PBMC cultures alone in both RA patients and healthy individuals (P < 0.001 and P < 0.05, respectively). IFN-γ stimulation of DFSCs further increased FoxP3 expressing Treg cells in RA patients (P < 0.0001). This data suggest that DFSCs can regulate immune responses by increasing the amount of FoxP3 expressing Treg cells (Figure 4).

### 3.5. DFSCs regulated inflammatory response by decreasing TNF-α and increasing IL-10 levels in RA patients

**Figure 5:**
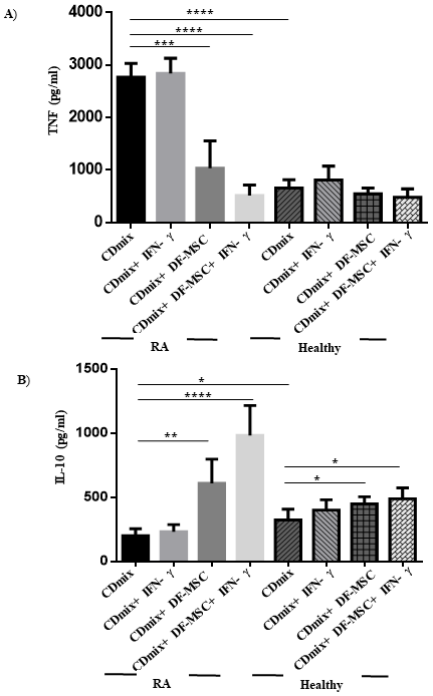
Cytokine levels in culture supernatants. TNF-α level was high and IL-10 levels were low in CDmix stimulated PBMC cultures of RA patients compared to healthy individuals. DFSCs significantly decreased TNF-α levels in CDmix (P < 0.001) and IFN-g stimulated (P < 0.0001) cocultures in RA patients. DFSCs increased IL-10 levels in CDmix (P < 0.01) and IFN-g stimulated (P < 0.0001) cocultures in RA patients. *P < 0.05, **P < 0.01, ***P < 0.001, ****P < 0.0001. Results are shown as mean ± SD.

To assess the immunomodulatory effects of DFSCs on lymphocyte phenotypes we evaluated proinflammatory and antiinflammatory cytokine levels in PBMCs of RA patients. Both cytokines were analyzed in PBMC cultures in the presence and absence of DFSCs. PBMC cultures of RA patients showed high levels of TNF-α, which is the signature cytokine of inflammatory responses in lymphocytes, and lower levels of IL-10 compared to healthy individuals (P < 0.0001 and P < 0.05, respectively). TNF-α levels were significantly decreased (P < 0.001) and IL-10 levels were significantly increased (P < 0.01) when cultured with DFSCs compared to PBMC cultures alone in RA patients, whereas no significant change in cocultures was observed in TNF-α levels in healthy individuals (P ˃ 0.05). IFN-γ stimulated cocultures decreased TNF-α levels compared to cocultures without IFN-γ stimulation in RA patients (P < 0.0001). IFN-γ stimulated cocultures also increased IL-10 levels compared to cocultures without IFN-γ stimulation in RA patients (P < 0.0001) (Figure 5).

## 4. Discussion

In this study, we showed that DFSCs suppressed the proliferation of activated lymphocytes of RA patients by increasing the frequency of CD4+CD25+Foxp3+ T regulatory cells. In addition, a significant reduction of PBMC apoptosis was observed in the presence of DFSCs in RA patients.

Rheumatoid arthritis is a chronic autoimmune disease that causes progressive articular damage and functional loss of joints. The development of new antiinflammatory drugs and kinase inhibitors in the past two decades has substantially improved clinical outcomes. Cytokine inhibitors have definitively proven the critical role of TNF-α and IL-6 in disease pathogenesis. Many patients can achieve long remissions if the disease is recognized and treated promptly and continuously; however, some individuals do not respond adequately to treatment. Nonetheless, no therapy is curative and clinical remission does not necessarily correspond to nonprogression of joint damage.

In this context, mesenchymal stem cells (MSCs) are potential candidates for repairing damaged tissues in joint disorders, including RA. Additionally, MSCs with the ability to differentiate into cartilage are present in joint tissues and raise an opportunity for therapeutic interventions via targeting intrinsic repair mechanisms. In addition to the ability to differentiate into cartilage or other tissue cells, MSCs can interact with the immune system and play an active role in the regulation of arthritis and progression of joint damage.

To examine the T cell activation in RA pathogenesis, we investigated the proliferative responses of T cells in the presence of DFSCs. According to our results, DFSCs decrease pathogenic T cell responses in RA patients. In agreement with our data, MSCs isolated from different tissues (bone marrow or adipose tissue) were previously shown to reduce inflammatory and T cell responses and induce regulatory T cells in vitro in RA (16,17). However, an important issue for clinical translation of MSCs for RA treatment is the requirement of large number of cells for infusion during therapeutic uses. However, recent studies demonstrated that large amounts of DFSCs can be easily obtained from follicle tissues of healthy donors and rapidly expanded in vitro to generate a clinically effective dosage.

A key role of aberrant pathways of T cell activation in the initiation and/or progression of RA is suggested. Previous studies showed that MSCs not only show immunomodulatory capacity, but also have antiapoptotic properties that cause supporting activity toward different cell types under resting conditions (19,20). In our previous study, we showed that DFSCs decreased the expression of apoptosis-related ligand and receptors on healthy individuals’ lymphocytes (21). In the present study, we investigated the effects of DFSCs on cell survival of T cells of RA patients. Lymphocyte viability ratio was low in CDmix stimulated PBMC cultures of RA patients, while it was high in healthy controls. DFSCs significantly increased T cell viability and decreased apoptosis in CDmix stimulated cocultures in RA patients. IFN-g stimulated DFSCs further increased the viability of T lymphocytes in RA patients. DFSCs also had an antiapoptotic effect on T cells of RA patients, and IFN-g stimulation increased this effect of DFSCs.

Treg cells play a central role in controlling autoimmunity, and their dysfunction leads to immune activation. A number of studies suggested that stem cell or biological therapies induce a potent population of CD4+CD25+ Treg cells in patients with RA (22,23). The current study revealed that low levels of CD4+CD25+Foxp3+ regulatory T lymphocyte population were increased with DFSCs in RA patients, associated with elevated IL-10. IFN-g stimulated DFSCs further enhanced CD4+CD25+Foxp3+ regulatory T lymphocyte frequency. Therefore, induction of CD4+CD25+Foxp3+ T regulatory cells could significantly contribute to the suppressive activity of DFSCs on RA T cells.

As a critical cytokine driving inflammation, TNF-α is a key therapeutic target for RA treatment (24). A previous study demonstrated that conditioned medium MSC treatment prevents TNF-α rise in sera from arthritic mice while it increased IL-10 levels (25). In accordance with this data, DFSCs suppressed the expression of TNF-α levels and increased IL-10 expression in RA patients in our study. IFN-g stimulated DFSCs further enhanced immunosuppressive effects on cytokine levels in RA patients.
